# Chemical Treatments Tested Against *Xylella fastidiosa*: Strategies, Successes and Limitations

**DOI:** 10.3390/pathogens14090840

**Published:** 2025-08-23

**Authors:** Letizia Portaccio, Marzia Vergine, Alessandro Bene, Mariarosaria De Pascali, Erika Sabella, Luigi De Bellis, Andrea Luvisi

**Affiliations:** 1 Department of Biological and Environmental Sciences and Technologies, University of Salento, 73100 Lecce, Italy; letizia.portaccio@unisalento.it (L.P.); alessandro.bene@unisalento.it (A.B.); mariarosaria.depascali@unisalento.it (M.D.P.); erika.sabella@unisalento.it (E.S.); luigi.debellis@unisalento.it (L.D.B.); andrea.luvisi@unisalento.it (A.L.); 2 National Biodiversity Future Center, 90133 Palermo, Italy

**Keywords:** control strategies, bacteria, *in vivo*, *in vitro* experiments

## Abstract

*Xylella fastidiosa* (*Xf*) is a Gram-negative bacterium responsible for severe diseases in several commercially significant crops, including olive, grapevine, citrus and almond. Its management is particularly challenging due to its transmission via widespread vector insects, its ability to form biofilms, its high genetic diversity and, sometimes, latent symptoms. Current control strategies focus on integrated and preventive approaches, including the use of resistant varieties, agronomic practices, and vector control through chemical and biological methods. Direct control of the bacterium has always been a complex challenge that includes strategies to limit vector presence and activity in the field; however, several compounds have recently been evaluated that are able to inhibit biofilm formation and *Xf* growth. This review provides an up-to-date summary of studies investigating the efficacy of various treatments based on organic compounds, synthetic molecules and salt- or metal-based formulations. By evaluating the results of *in vitro* and *in vivo* experiments, the most promising solutions were identified that address the main challenges and limitations of chemical control strategies. These include N-acetylcysteine and zinc- and copper-based formulations, which are effective and potentially transferable to the field for crops such as citrus and olive trees. Antimicrobial peptides and nanoparticles, on the other hand, have demonstrated high efficacy *in vitro*, although further studies directly in the field are required. The evidence emerging from the analyzed studies offer insights to guide future research towards more effective and sustainable management approaches to mitigate the spread and impact of *Xf*.

## 1. Introduction

*Xylella fastidiosa* (*Xf*) is a Gram-negative bacterium belonging to the Xanthomonadaceae family, with the ability to colonize the xylem vessels of numerous host plants. Vector insects, such as spittlebugs and leafhoppers, are responsible for transmission as they feed on raw sap and spread pathogens from an infected plant to a healthy one [1]. The bacterium’s ability to survive in various hosts and environments, along with its transmission mode, makes its management complex.

*Xf* settles in the xylem vessels once the infection has occurred, where it adapts to a nutrient-poor environment by forming biofilms that progressively obstruct the flow of water and nutrients. As a result, affected plants exhibit symptoms such as leaf desiccation, reduced growth and death [2]. The severity of the disease depends on the host species and environmental conditions, causing severe damage to crops such as olive trees, grapevines, citrus and almond trees [3,4].

One of the most critical aspects of *Xf* is its genetic and biological diversity, being classified into several subspecies—including *pauca*, *fastidiosa*, *multiplex* and *sandyi* [5,6]—and it is capable of infecting more than 570 plant species (EPPO Global Database https://gd.eppo.int/taxon/XYLEFA/hosts, available online 11 August 2025). This genetic diversity makes management further complex, as variants of the bacterium can exhibit different behaviors towards host plants [7]. Currently, control strategies are based on integrated and preventive approaches, such as using resistant or tolerant varieties, targeted agronomic practices, weed control and soil management.

In recent years, several studies have focused on identifying chemical molecules that are potentially effective in controlling *Xf*. Research has examined a broad spectrum of compounds, evaluating their ability to inhibit bacterial growth and reduce biofilm formation. The main groups of substances analyzed include compounds of organic origin, synthetic molecules (including antibiotics and metal derivatives) and formulations based on salts and metals. Although the use of genetic means and the chemical control of insect vectors are crucial components in *Xf* management, this review aims to provide a comprehensive and updated overview of the direct efficacy of various compounds in *Xf* control. These compounds are analyzed in two experimental settings: *in vitro* experiments, in which they are tested on isolated bacterial cultures to determine their antibacterial action, and *in vivo* studies, which evaluate the impact of treatments on infected plants. Through this systematic analysis of the results of different experimental approaches, the aim is to identify the most promising solutions for effective *Xf* management and to highlight the main challenges and opportunities associated with the use of these chemical strategies in *Xf* control. Finally, a brief compendium on the control of some main vectors by chemical means is reported, where the strategy currently used to prevent the spread of the insect is examined, including the use of systemic or contact insecticides and treatments based on mineral oils.

The data reported in the literature aim to reflect the multiple challenges posed by this bacterium and to articulate a path towards practical and sustainable agricultural solutions. Furthermore, continued research is essential to adapt and evolve management strategies that can address the rapidly changing agricultural landscape characterized by the presence of *Xf*.

## 2. Organic Molecules

Chemical compounds, such as organic molecules, are essential for many biological and chemical processes. Some of these molecules have antimicrobial and antioxidant properties necessary for improving plant health and increasing their resistance to biotic and abiotic stresses, which is why they are utilized in crop protection.

### 2.1. N-Acetylcysteine (NAC)

N-acetylcysteine (NAC) is a derivative of cysteine with numerous therapeutic properties, usually used in the medical field [8,9]. It is suitable for many uses due to its multifaceted roles, including as an antioxidant, a disulfide bond disruptor and a metal chelator [10,11]. It is used to combat oxidative stress, serving as a precursor to glutathione and reducing the damage caused by reactive oxygen species (ROS) [12,13].

From an antimicrobial perspective, NAC is effective because it reduces the formation of biofilms by many Gram-negative and Gram-positive bacteria. This occurs through the interruption of the extracellular polysaccharide matrix, thus promoting the reduction of bacterial aggregates and the breakdown of biofilms. Thanks to these properties, NAC is also used as a non-antibiotic alternative, helping to limit bacterial resistance [14,15].

Finally, its characteristics, including antibacterial properties, the ability to inhibit biofilms, low cost and the absence of significant environmental impacts, make NAC a promising candidate for testing against *Xf* [16]. This has led to research being carried out *in vitro* and *in vivo*.

Muranaka et al. [16] performed *in vitro* experiments with the bacterial strain 9a5c, isolated from sweet oranges suffering from Citrus Variegated Chlorosis (CVC) due to subsp. *pauca*, which was treated with different concentrations of NAC (1.0–6.0 mg/mL) for 14 days, analyzing biomass, cell viability and biofilm formation. The results showed that NAC inhibited biofilm formation and the production of exopolysaccharides (EPS) while exhibiting antimicrobial properties. At concentrations above 1.0 mg/mL, a significant reduction in biofilm formation was observed, accompanied by an increase in planktonic cells, which were primarily dead, indicating the antimicrobial effect of NAC. Cell viability was assessed using an aliquot of the affected fraction diluted in phosphate-buffered saline with the subsequent counting of colony-forming units. Finally, microscopic analysis confirmed the inhibition of cell adhesion and the lack of a structured biofilm at higher concentrations [16]. The finding was also confirmed by Da Silva et al. [17] on *Xf* strain 11 339 extracted from sweet orange trees, but to learn more about the effects of NAC on EPS, force spectroscopy measurements (a technique that allows one to analyze the cellular adhesion on live cells in conditions similar to real ones [18]) were conducted on treated and untreated samples to such an extent that it was observed that in those treated with NAC, the residual biofilms on the surface were less dense than those observed in the untreated sample. The morphology of the individual treated cells also differed, with some exhibiting a more irregular surface [17]. On the contrary, in the research conducted by Cattò et al. [19], on olive trees affected by the *Xf* subsp. *pauca* strain De Donno and treated with NAC, the results showed that 1000 μM of NAC did not affect bacterial growth, whereas a lower concentration (500 μM) reduced adhesion, increased biofilm biomass and favored the formation of cell clusters. Furthermore, NAC increased oxidative stress and reduced biofilm dispersion without altering cell viability [19].

Therefore, despite the antimicrobial and antibiofilm properties highlighted by the studies of Muranaka et al. [16] and da Silva et al. [17], it is necessary to underline that the efficacy of NAC varies according to the host and bacterial strain.

In support of *in vitro* observations, *in vivo* studies were conducted on infected plants under different experimental conditions. The study conducted in a hydroponic system by Muranaka et al. [16] examined the efficacy of NAC (at concentrations of 0.48, 2.4 and 6 mg/mL) in treating CVC-infected sweet orange plants. The plants, obtained by grafting infected branches onto “Rangpur” lime rootstocks, were grown in a greenhouse for two years, during which time they developed CVC symptoms on all new branches. The infected plants were treated with NAC by fertirrigation plus trunk injection. The latter has been used because it offers several advantages compared to traditional soil irrigation with pesticides, including greater operator safety, precision in product application and greater attention to non-target organisms [20]. On the other hand, it is a technique that is little used in agriculture because of its high cost, and therefore, it is not accessible to farmers [21]. The results of the study by Muranaka et al. [16] showed that treatment with NAC significantly reduced the bacterial load and mitigated the symptoms of CVC. In addition, a slow-release fertilizer containing NAC was tested, which prolonged the therapeutic effect and slowed the onset of symptoms. Treated plants exhibited a reduction in symptom severity and bacterial overgrowth compared to untreated controls; however, discontinuation of treatment led to the reappearance of symptoms, indicating that continuous treatment is necessary for effective control. For both plants with few initial symptoms and plants with advanced symptoms, NAC injection into the trunk did not enhance the result already obtained previously with fertirrigation alone, and this is why treatment with fertirrigation alone is sufficient. NAC, however, had no adverse effects on healthy plants. In conclusion, treatments with NAC, either as a nutrient solution or with slow-release fertilizers, show potential for CVC control; however, efficacy depends on continuous administration [16].

Following the evaluation of NAC efficacy in greenhouse conditions, short-term and long-term field research was conducted on sweet orange and pear plants affected by CVC to assess NAC translocation. After the various applications through fertilization, it was shown that in the short-term experiment (a single application of NAC), the NAC distribution was 89% in the roots, 5.3% in the stem and 1.9% in the leaves, unlike the long-term experiment (application of NAC every two weeks for three months), where the accumulation of NAC was 69.8% in the roots, 11.2% in the stem and 19% in the leaves. Thus, the increase in applications led to the rise in concentration in epigeal plant organs and reduced the number of blocked xylem vessels. To determine if the treatment could affect the flow of sap, the transpiration rate was measured, showing that it was highest for healthy plants (a peak of 2.54 mmol/m^2^ s at 12:00 h). In plants affected by CVC, the peak was 0.97 mmol/m^2^ s at 12 h, and there was also a decrease in oxidative stress, as evidenced by the improvement in the activity of detoxifying enzymes. NAC has also been crucial in enhancing fruit yield in diseased plants [22].

A further study by Saponari et al. [23] evaluated NAC’s effectiveness in olive trees affected by *Xf*, showing a partial reduction in symptoms under certain conditions. Notably, symptom relief was only visible at low bacterial titers, highlighting the complexity of NAC’s interactions with both host plants and the pathogen. The phytotoxicity observed at higher NAC concentrations requires careful dose management, as excessive applications may cause adverse effects, such as leaf drop [23]. The reported results underline the efficacy of NAC both *in vitro* and *in vivo* due to its antibiofilm and antibacterial activity, but its application is strongly conditioned by the type of administration, the host and the bacterial strain. Unlike in some cases where there was a reduction in the bacterial titer and an improvement in the conditions of the plant, in other cases—such as for olive trees infected by *Xf* subsp. *Pauca*—the efficacy of NAC was limited, as it showed a partial reduction in symptoms and because it was only valid for plants with an initially low bacterial titer.

The mechanistic insights into NAC’s action, particularly in mitigating oxidative stress responses and disrupting biofilm formation, offer valuable clues for optimizing its use in agricultural settings. NAC is a versatile therapeutic agent with promising applications in both *in vivo* and *in vitro* farming environments. Its various roles as an antioxidant, a biofilm disruptor and an antimicrobial agent demonstrate a significant range of effectiveness against different biological challenges. However, despite its proven potential, the variability in the results depending on concentration, host plants and bacterial strains calls for a careful approach to its application. Ongoing research to understand how NAC works, combined with rigorous field trials, will be key to unlocking its full therapeutic capabilities against plant pathogens such as *Xf*, while also supporting wider efforts for agricultural sustainability.

### 2.2. Phenolic Compounds

Plants produce phenolic compounds as a defensive strategy against various pathogens, including bacteria, fungi and viruses [24]. Several studies have demonstrated that these compounds, either alone or in combination, can inhibit the growth of various bacterial species [25,26]. In particular, it has been reported that some strains of *Xf* affecting grapevines and almonds show reduced growth *in vitro* in the presence of specific phenolic compounds, such as hydroxytyrosol, tyrosol, catechol, 4-methyl catechol, oleuropein, verbascoside, 4-hydroxybenzoic acid, vanillic acid and p-coumaric acid [27].

Comprehensive experiments employing dilution methods on agar plates established the minimum inhibitory concentrations (MICs) for twelve different phenolic compounds against subsp. *fastidiosa* strains (Temecula and Conn Creek) and subsp. *multiplex* strains (Dixon and Tulare), revealing that several phenolic acids, including gallic and caffeic acids, catechol, rutin and resveratrol, exhibited potent antibacterial activity, often with stronger inhibitory effects than those reported against Gram-positive and Gram-negative bacteria in previous studies [28]. Compounds with simpler structures, such as catechol and coumarin, have demonstrated bactericidal and fungicidal effects [29]. In contrast, flavonoids like naringenin and catechin, along with stilbenes like resveratrol, have shown strong inhibitory activity attributed to their ability to disrupt microbial cell integrity. The antimicrobial efficacy of phenolic compounds against *Xf* subsp. *fastidiosa* is attributed to several mechanisms, including interference with bacterial adhesion, a crucial step in biofilm formation. Phenolic compounds such as gallic acid and epicatechin have been shown to reduce bacterial adhesion to surfaces and negatively regulate the expression of genes associated with adhesive structures, such as *fim*A and *xad*A [30]. This inhibition of biofilm formation is crucial, as biofilms significantly contribute to bacterial resistance against antimicrobial treatments. Furthermore, a study by Vizzarri et al. (2023) [31] examined the antibacterial properties of olive leaf extracts containing notable phenolic compounds, such as oleuropein, showing that these formulations significantly inhibited *Xf* growth and biofilm formation. Notably, oleuropein was identified as particularly potent and promising as a natural anti-*Xf* agent.

While *in vitro* studies confirm the potential of phenolic compounds in managing *Xf*, their practical application in the field faces challenges related to absorption and systemic distribution within plants. Research on olive trees infected by *Xf* subsp. *pauca* indicated that, although treatments showed some efficacy, the overall absorption rates in mature trees were inadequate due to xylem obstruction caused by the pathogen [32]. To address this limitation, the use of potassium phosphite in conjunction with phenolic treatments has been proposed, as it enhances vegetative growth and facilitates compound uptake, suggesting a biostimulatory effect that may improve treatment outcomes [31].

In conclusion, most of the experiments were conducted *in vitro*, highlighting the efficacy of some phenolic compounds in inhibiting the bacterial growth and biofilm formation of *Xf fastidiosa* and *multiplex* subspecies. Regarding *in vivo* tests, conducted to control *Xf* subsp. *pauca* in olive trees, we know very little about the post-treatment bacterial titer, highlighting that the uptake of phenolic compounds was limited, especially in adult trees, thus compromising their efficacy. However, the difficulties related to uptake and practical application in the field require further research aimed at optimizing the methods of administration and formulations to allow for the effective use of these natural compounds in sustainable agricultural practices.

### 2.3. Oxylipins

Lipids, which are derived from plant and animal organisms, play a crucial role in biological functions, serving as an energy source and signaling molecules, including hormones. These compounds primarily consist of fatty acids or their derivatives, and their composition varies depending on the type of plant lipids; specifically, they comprise a mixture of polar and non-polar lipids, as well as acylglycerides, which serve as reserves [33]. Lipids include oxypilins, which could represent a control strategy against *Xf*.

The research conducted by Scala et al. [34] highlighted the differential lipid composition between olive trees infected by *Xf* subsp. *pauca* (cv. Ogliarola) and healthy counterparts. By analyzing symptomatic and asymptomatic plants, the study revealed that certain oxylipins and unsaturated fatty acids were more abundant in infected samples. Specifically, the lipoxygenase-derived oxylipins were found to promote biofilm formation, while dioxygenase-derived oxylipins inhibited it, elucidating their role in bacterial virulence regulation. This study aligns with previous findings, which indicate that symptomatic trees exhibit exaggerated lipid accumulation compared to non-symptomatic trees [35]. Scala et al. [36] demonstrated that several specific oxylipins, such as 9-hydroxy-10,12,15-octadecatrienoic acid, have a pronounced effect on the biofilm formation of *Xf*.

In contrast, compounds like 7,10-dihydroxy-8-octadecenoic acid have been shown to inhibit biofilm development, highlighting the dual nature of oxylipins in modulating bacterial behavior. Increased biofilm formation correlates with disease severity, suggesting that oxylipins may exacerbate *Xf* infections by facilitating the establishment of biofilms within host tissues. Recent investigations into the mutations of specific genes related to *Xf* virulence have shed light on the possible regulation of oxylipin production. Scala et al. [37] analyzed the impact of the PD0744 gene mutation on oxylipin dynamics and biofilm formation in *Xf* subspp. *fastidiosa* and *multiplex* strains Temecula1 and AlmaEM3. The results indicated that the mutant strains exhibited a marked reduction in biofilm formation, which could be attributed to a lack of the *Xad*A2 protein known to facilitate adhesion. Interestingly, while the planktonic growth of the mutant strain was greater, the biofilm inhibitory effects raise compelling questions regarding the balance of pathogenicity and dormancy in bacterial populations under the influence of oxylipins [37].

The presence of oxylipins has a significant impact on the interaction between *Xf* and host plants. Understanding the mechanisms by which these compounds influence biofilm formation and bacterial growth is crucial for developing new disease control strategies, as these compounds are not yet established as practical treatments. The dual roles that certain oxylipins play—promoting both virulence and inhibiting adhesion—indicate potential molecular targets for agricultural interventions aimed at mitigating the effects of *Xf* on economically significant crops.

However, it is crucial to note that the majority of studies conducted so far have been *in vitro*, which raises concerns regarding the practical transferability of these findings to real-world agricultural settings. Future research should focus on translating laboratory insights into field applications, establishing protocols that leverage targeted oxylipin manipulation to disrupt *Xf* infection cycles effectively.

### 2.4. NuovOlivo^®^

Recently, trials were conducted with a product called NuovOlivo^®^, a natural detergent based on vegetable oils and an aqueous infusion of various plants combined with sodium hydroxide, calcium and sulfur. Its composition includes typical Mediterranean plants such as *Thymus vulgaris* L., *Petroselinum crispum* (Mill.) Fuss, *Crataegus monogyna* Jacq., *Rosmarinus officinalis* L., *Salvia officinalis* L., *Origanum vulgare* L., *Matricaria chamomilla* L., *Malva sylvestris* L., *Salix babylonica* L., *Capsicum annuum* L. and *Piper nigrum* L. [38]. Trials in olive trees infected by *Xf* subsp. *pauca* were carried out at two sites in Apulia (Italy), involving cultivars Cellina di Nardò and Ogliarola salentina, both susceptible to *Xf*. While treated plants showed some signs of recovery, such as new growth, flowers and fruit production, and a reduction in bacterial DNA content was observed (up to 99.04% reduction in some cases), the trials were conducted under limited controlled conditions and involved limited sample sizes [38]. Infection indices decreased significantly in treated plants compared to the controls, yet the extent of disease control may vary depending on environmental factors, tree age and the severity of infection. Additionally, pruning did not consistently demonstrate significant synergistic effects alongside treatment. The treatment also appeared to improve physiological parameters, such as leaf size and phenolic content, and reduce indicators of cell damage, but longer-term effects and the durability of these improvements remain uncertain. A further study in 2024 expanded the treatment to several olive cultivars across multiple groves, with treated plants showing better vegetative growth and symptom reduction compared to the untreated controls; however, variability in the results was notable across cultivars and sites [39]. While these findings are promising and suggest a potential role for NuovOlivo^®^ in integrated disease management, the limited scale and experimental nature of the studies warrant cautious interpretation. Considering that the current results on NuovOlivo^®^ are based on preliminary studies with limited sample sizes and without independent replications, larger field trials, over multiple growing seasons, are needed to fully evaluate the efficacy, optimal application protocols, and economic feasibility of these treatments before recommending their large-scale adoption.

## 3. Synthetic Molecules

Some products used in agriculture are of synthetic origin because they are obtained through non-natural processes [40] and still account for the predominant share of the total active substances in the EU compared to those of natural origin [41]. Still, despite their importance, safer and more sustainable alternatives need to be explored.

### 3.1. Menadione and Benzethonium Chloride

Vitamin K comprises a group of lipophilic vitamins, with vitamins K1 and K2 representing the natural forms typically found in green leafy vegetables and produced by intestinal bacteria, respectively. Menadione, or vitamin K3, is the synthetic variant that lacks the polyisoprenoid side chain characteristic of its natural counterparts [42]. Conversely, benzethonium chloride is a quaternary ammonium salt recognized for its antimicrobial properties against specific bacteria and fungi [43]. In an experimental study by Zhang et al. [44], the efficacy of menadione and benzethonium chloride was evaluated both *in vitro* and *in vivo* for their potential to combat Pierce’s disease. The study involved the genetically modified Temecula 1 strain of *Xf* subsp. *Fastidiosa*, which expresses green fluorescent protein, to facilitate the measurement of bacterial proliferation. *In vitro* results indicated that both menadione and benzethonium chloride at concentrations of 25, 50 and 100 μL achieved 100% inhibition of bacterial growth. However, the phytotoxicity assessments revealed detrimental effects on plant health; benzethonium chloride caused chlorophyll loss proportional to the concentration (approximately 33% at 100 mM), while menadione led to an immediate loss of 40–45% chlorophyll across all tested concentrations. The *in vivo* treatments included foliar sprays and soil irrigation. Menadione applied through foliar spraying induced necrosis in leaves, whereas benzethonium chloride treatments were less aggressive. Notably, menadione’s phytotoxicity decreased when applied via soil irrigation, while benzethonium chloride showed minimal phytotoxicity as a foliar spray. Despite these issues, both treatments effectively mitigated disease progression in treated grapevines; after three months, treated plants showed symptoms in about 50% of nodes compared to complete defoliation in untreated controls [44]. Overall, the results indicate that in vitro, menadione and benzethonium chloride completely inhibit the growth of *Xf* subsp. *fastidiosa* with variable phytotoxicity depending on the molecule and the administration method. *In vivo*, however, there was no direct quantification of the bacterial titer, but the treatments have been shown to slow down the disease on grapevine.

### 3.2. Nanoparticles

Nanotechnology is a crucial tool in modern agriculture, and one promising method to counter *Xf* appears to be the use of nanoparticles (NPs). To date, all those materials with a size of 1 to 100 nm are defined as such [45], which in agriculture are called “nanopesticides” [46]. In particular, metal nanopesticides exhibit antimicrobial activity *in vitro* and protect plants against bacterial diseases [47,48]. 

Metallic nanopesticides can persist in the environment, accumulate in soil and non-target organisms and, if used uncontrolled, promote the development of antimicrobial resistance, especially with frequent and high-dose applications [46].

Among the various types of nanopesticides, some have demonstrated exceptional effectiveness. The main nanoparticle-based treatments investigated for their efficacy against *Xf* are summarized in the table below (Table 1), highlighting their mechanisms of action, key characteristics and relevant references.

#### 3.2.1. Silver (Ag) NPs 

Orfei et al. [65] examined the potential of silver (Ag) nanoparticles to prevent biofilm formation across multiple bacterial strains, including three Gram-negative species (*Pseudomonas syringae*, *Xanthomonas vesicatoria* and *Xf* subsp. *pauca*) and one Gram-positive species (*Clavibacter michiganensis*). Treatment with Ag nanoparticles at concentrations between 0.1 and 1.0 ppm (incubated for 24–48 h) led to a significant reduction in biofilm formation, with up to a 97% inhibition across all tested strains. Notably, even at the lowest effective concentrations (0.06 ppm for *X. vesicatoria* and 0.023 ppm for *Xf*), substantial inhibition was observed, underscoring the strong potential of low-dose nanoformulations for managing agricultural diseases [65].

#### 3.2.2. Thymol Nanoparticles

Building upon this research, Baldassarre et al. [66] investigated the use of thymol nanoparticles against *Xf* subsp. *pauca*. To overcome thymol’s limited water solubility, they employed calcium carbonate nanocrystals to improve its bioavailability and antibacterial activity. Treatments with both free thymol and thymol-loaded nanoparticles demonstrated notable antibacterial effects, with the nanoparticle formulation at concentrations of 0.25 and 2 mg/mL showing a more potent inhibition of bacterial growth. These findings highlight the advantages of nanoencapsulation strategies in enhancing the efficacy of hydrophobic natural antimicrobials [66].

Extending these findings to field conditions, Cagnarini et al. [67] conducted *in vivo* trials on 40 olive trees infected with *Xf* subsp. *pauca* in different locations, comparing a 3% thymol solution to a formulation containing thymol encapsulated in cellulose nanoparticles. Although the nanoparticle-based treatment showed a slight reduction in bacterial loads over time, statistical analysis indicated no significant differences compared to the free thymol treatment. This suggests that further investigation into long-term effects and efficacy under various environmental conditions is necessary [67]. 

#### 3.2.3. Fosetyl–Al Nanocrystals

Additionally, Baldassarre et al. [68] evaluated Fosetyl–Al nanocrystals (NanoFos), produced via sonication and subsequently coated with Chitosan–Fosetyl–Al nanocrystals (CH–nanoFos), for antibacterial activity against *Xf* subspecies. The formulation completely inhibited the growth of *Xf* subsp. *fastidiosa* at 100 μg/mL after six days and reduced biofilm formation by 50–60% after 15 days. Conversely, its effect on *Xf* subsp. *pauca* was more moderate, with only the partial suppression of planktonic growth and minimal impact on biofilm formation, indicating the efficacy of strain-dependent variability [68]. A recent *in vivo* study conducted on *Nicotiana tabacum* demonstrated that CH–nanoFos was able to significantly reduce bacterial colonization by *Xf* subsp. *Fastidiosa*, *multiplex* and *pauca* inhibit the formation of biofilm but also limit the development of visible symptoms [69].

Other nanotechnologies, such as hyaluronic acid–chitosan nanofilms at different pHs, were tested on *Xf* subsp. *pauca* to evaluate their physicochemical and antibacterial properties. At pH 4.5, both prepared nanofilms showed significant antibacterial activity. Increasing the ionic strength did not substantially change the antibacterial effect, but it significantly influenced the physicochemical properties of the films, including film thickness, the amount of deposited polymer and surface morphology and topography. At pH 3.0, the nanofilm showed the best antibacterial effect. This was attributed to the increased exposure of protonated ammonium groups (NH_3_^+^) on the chitosan surface. These highly positively charged groups interact with the negatively charged bacterial membranes, causing cell disorganization and death [70].

The current literature highlights the significant potential of nanoparticle-based strategies for *Xf* control. While *in vitro* studies consistently show strong antibacterial and antibiofilm activities, their translation to *in vivo* settings has yielded more variable and sometimes limited results, as data on bacterial titer are limited or show insignificant reductions, as in the case of thymol nanoparticles. Furthermore, the latter are studied in olive trees, hosts of *Xf* subsp. *pauca*, while for the other subspecies, no studies on species of agricultural interest are available. This highlights the need for further in-depth research focused on optimizing formulations, evaluating their performance in different cropping systems and environmental conditions, and assessing long-term impacts on plant health and ecosystems. Integrating nanotechnology into plant protection strategies is a promising approach to increase resilience against emerging bacterial threats; however, its success will depend on careful refinement and validation to ensure both efficacy and sustainability in agricultural practices.

#### 3.2.4. Zinkicide^®^

Zinkicide^®^ (ZnK) is a liquid antimicrobial nanoformulation with ZnO nanoparticles of about 4 nm that is effective against certain bacteria [59]. The antimicrobial activity encompasses the production of reactive oxygen species, lipid peroxidation, cell membrane disruption and the accumulation of Zn ions in the solution [59,60,61]. Moreover, it has an eight times lower minimum inhibitory concentration than other bactericides, such as Cu-based bactericides [60].

ZnK was used in research by Shantharaj et al. [71] to understand whether ZnK can eliminate the bacterium or reduce its growth. *In vitro*, a strain of *Xf* subsp. *fastidiosa* (TemeculaL) was grown on agar plates for two weeks and then suspended in phosphate-buffered saline. Next, a minimum bactericidal concentration test was performed by growing the bacteria in a liquid medium with different concentrations of ZnK and counting the number of surviving colonies. To simulate the natural environment of the plant, microfluidic chambers were used to mimic sap flow, where only the culture medium flowed in one channel and the ZnK medium flowed in the other. After one week, live cells were counted from the liquid flowing out of the chamber to verify whether the treatment was effective under real-world conditions. The results of the *in vitro* experiment showed that 50 ppm ZnK reduced the bacterial population by 99.9% within 1 h and promoted the inhibition of biofilm formation. In other bacteria, the efficacy of ZnK varies; for example, *Escherichia coli* is inhibited at 31 ppm, while *Xanthomonas* is inhibited at 62.5 ppm.

*In vivo*, *Nicotiana tabacum* and *Vaccinium corymbosum* plants were grown in a greenhouse at a controlled temperature and under regular fertilization. Subsequently, *N. tabacum* plants were inoculated with *Xf* subsp. *fastidiosa* (strain TemeculaL), while *V. corymbosum* plants were inoculated with *Xf* subsp. *multiplex* (strain AlmaEm3), and they subsequently received different concentrations of ZnK through fertirrigation every week for 4 weeks. Afterwards, several parameters were evaluated, including phytotoxicity, disease severity with symptom monitoring, mineral concentration, nutritional balance and the bacterial population of the plant, to understand if ZnK altered the nutritional balance of the plant. In *N. tabacum*, three treatments at higher doses (500 ppm, 500 ppm and 1000 ppm) led to the complete elimination of the bacterium and a reduction in the severity of *Xf* symptoms by 76%, but the dose at 1000 ppm caused stunted growth and the death of the plant with the consequent significant increase of Zn in the leaves and reduction of Ca, Mg and Mn.

In *V. corymbosum*, the situation was different because high doses of ZnK did not cause visible phytotoxicity, and this led to the administration of even higher doses (1000 ppm, 1000 ppm and 500 ppm), which significantly reduced the bacterial population by about 2 log_10_ and reduced the severity of symptoms by 43%; however, at the nutritional level, the plants had a lower capacity for Zn accumulation in the leaves [71].

Finally, ZnK has shown high efficacy *in vitro* in reducing bacterial titer in just 60 min and inhibiting biofilm formation. *In vivo*, studies have been conducted only on model plants such as *N. tabacum* and *V. corymbosum*, where ZnK has allowed for a reduction of symptoms of 76% and 43%, respectively, highlighting that high doses of ZnK can cause nutritional imbalances and phytotoxicity.

#### 3.2.5. Calcium Carbonate Nanocarriers

Calcium carbonate (CaCO_3_) is considered a cheap and versatile material used in various research fields. Its properties depend on several characteristics, including particle size and crystalline phase. For this reason, the synthesis of CaCO_3_ nanoparticles and microparticles for agricultural use has become widespread in recent years [72]. Molecules and macromolecules can be integrated into carrier systems in different ways: by physical adsorption on the surface of nanoparticles, by chemically bonding with them or by enclosing them in nanometric structures [62,63,64].

Starting from these considerations, the study by Baldassarre et al. [73] represents an example of the experimental application of CaCO_3_ nanocarriers and involved mixing calcium chloride and sodium bicarbonate solutions to create CaCO_3_ nanocrystals, which were subsequently coated with a fluorescent dye. They were used to transport substances such as caffeic acid and NAC (loaded by physical adsorption by mixing the solutions containing pesticides with nanocrystals). The aim was to evaluate the interaction between the nanocrystals at different concentrations (1 mg/mL, 100 μg/mL, 10 μg/mL and 1 μg/mL) and *Xf* subsp. *pauca* (strain 9a5c). Olive petioles, twigs and cuttings were immersed in fluorescent nanocrystal solutions to test their absorption and translocation through the xylem tissues. Nanocrystals caused bacterial wall damage, membrane detachment and, in some cases, cell destruction. In addition, the presence of surfactants on nanoparticles (NPs) was crucial for the mechanism of toxicity and adhesion of biomolecules [74]. In *Xf*, the reduction in growth was 60–70% compared to the untreated controls. Another clear sign that the bacterium responded to the treatment was the production of membrane vesicles (associated with environmental interactions and defense mechanisms). This was also possible thanks to the loading of antimicrobial compounds (caffeic acid) in CaCO_3_ nanocrystals, which made the treatment more effective by allowing for a targeted delivery through the vascular system of plants to achieve a therapeutic effect, also reducing the amount of drug needed. From the analyses carried out, nanocrystals (about 90%) were detected in the xylem vessels, confirming their easy migration inside the plant, a crucial aspect of systemic therapy for infected plants.

Another objective of the research by Baldassarre et al. [73] was to test the efficacy of CaCO_3_ nanocrystals directly in olive trees infected by *Xf* subsp. *pauca*. They were initially used to demonstrate the ability of olive trees to absorb and translocate them and subsequently as vectors of some antimicrobial compounds (NAC and caffeic acid). Despite two months of treatment with nanovectors, the bacterial population remained unchanged (105 CFU/mL), suggesting that this system did not have a significant impact on the reduction of bacterial growth in treated plants [73].

The study showed that CaCO_3_ nanocrystals are effective only *in vitro* since they cause a significant reduction in the bacterial titer of *Xf* subsp. *pauca*, but in real conditions, where CaCO_3_ nanocrystals were tested on olive trees, they have a lack of efficacy since the bacterial titer remains unchanged. Furthermore, even at a symptomatic level, the olive trees after the treatment did not show any improvements.

### 3.3. Antimicrobial Peptides (AMPs)

Antimicrobial peptides (AMPs) are short sequences formed from cationic and hydrophobic amino acids, recognized for their antimicrobial properties and serving as innate defense mechanisms in both plants and animals [75,76]. AMPs are categorized based on their structural features, amino acid sequences and net charge, resulting in classifications such as anionic AMPs, helical α-cationic AMPs, extended cationic AMPs, β-sheet AMPs and antimicrobial protein fragments [77]. Additionally, their mechanisms of action can be delineated into non-porous models and transmembrane pore models, elucidating different pathways for antimicrobial activity. The cationic nature of AMPs allows them to interact favorably with the negatively charged components of bacterial membranes, promoting their binding and ultimately leading to cell disruption and death [78].

Given the relatively low toxicity of AMPs to host cells and their diminished potential for developing resistance, they have emerged as promising candidates for bacterial control, including efforts against the pathogen *Xf* [79]. Resistance development to AMPs occurs slower due to their positive charge and ability to form amphipathic structures that allow for interaction with negatively charged phospholipids on the surfaces of bacterial membranes [80].

**Figure 1:**
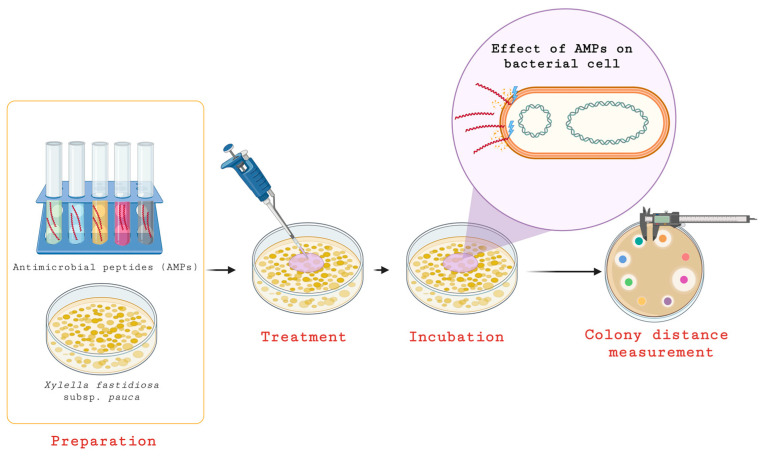
Evaluation of bacterial response to antimicrobial peptides (AMPs). According to the findings presented by El Handi et al. [81], antimicrobial peptides were administered to the *Xfp* bacterium at ascending concentrations (3, 50, 25 and 12 μM for *Xylella fastidiosa* subsp. *pauca*) to assess their impact on bacterial viability. Throughout the incubation period, these peptides exerted their action primarily by compromising the structural integrity of the bacterial membrane, leading to cell lysis and death. Post-incubation, the spatial dispersion of the bacterial colonies was systematically quantified by measuring the inter-colony distance, serving as a reliable indicator of the antimicrobial potency of the peptide treatments.

Research has evaluated the efficacy of various AMPs against *Xf* subsp. *pauca* and *Xanthomonas albilineans* by applying varying concentrations of AMPs and measuring their effects over time. The findings revealed a MIC range that varied across different peptides, with some AMPs emerging as particularly potent in reducing bacterial growth by notable percentages. Furthermore, these peptides have also been shown to inhibit biofilm formation and damage bacterial cells [81] (Figure 1).

To further assess the potential of AMPs against various *Xf* strains, studies have utilized techniques such as real-time quantitative PCR to quantify bacterial viability in the presence of specific peptides such as BP171, BP175, BP170, BP176 and BP180, along with the already known BP178, which significantly reduce bacterial viability, affirming their effectiveness [82]. Additionally, synthesized AMPs have exhibited variable efficacy, with certain peptides significantly reducing bacterial viability after treatment [83].

In a study by Li et al. [84], cecropin A, cecropin B, magainin I, magainin II and Shiva-1 were evaluated for their antimicrobial activity against *Escherichia coli*, *Agrobacterium tumefaciens* and *Xf* subsp. *fastidiosa*. Cecropin A and B showed the greatest efficacy, completely inhibiting *E.coli* growth at 0.5 µM as well as *Xf*, while Shiva-1 showed only partial inhibition at the highest concentration (10 µM) [84]. Instead, in the study by Moll et al. [83], the peptides were synthesized in vitro and tested at concentrations of 3.1 µM and 12.5 µM on *Xf* subsp. *fastidiosa* and *multiplex*. An evaluation of the results showed that the greatest bactericidal activity was provided by peptides such as RIJK2, 1036, Magainin 2 and Cecropin B. At 12.5 µM, peptide 1036 showed the highest activity (3.48 log reduction), surpassing Cecropin B (3.19 log). At 3.1 µM, RIJK2, 1036 and Cecropin B maintained a reduction between 1.89 and 2.13 log, while Magainin 2 showed very limited activity. RIJK2 was effective at all concentrations, while the other peptides showed a decrease in activity as the concentration decreased. Instead, the highest antibiofilm activity (80–90% reduction) was obtained by peptides such as 1026, RIJK2 and 1036 [83].

Gomesin, another antimicrobial peptide tested against the virulent strain *Xf* subsp. *pauca*, demonstrated strong activity, with effective concentrations noted [85]. However, its impact on biofilm formation suggested a complex interplay, as it appeared to increase biofilm formation in some cases, indicating the intricate nature of microbial interactions with AMPs [85].

*In vivo* evaluations have shown that treatment with AMPs post-infection can improve plant health compared to untreated controls, reporting reductions in bacterial populations and disease severity [81,86]. Nevertheless, some peptide treatments have resulted in mild phytotoxicity, signifying the necessity for careful dosing during application. Moreover, additional testing with gomesin illustrated that treatment at higher concentrations could reduce disease severity but remained insufficient for total infection control, highlighting that further optimization is needed for AMP applications against *Xf* [85].

AMPs present considerable promise for managing *Xf* infections, particularly *in vitro*, where they exhibit substantial antimicrobial and biofilm-inhibiting activity. Nonetheless, their *in vivo* efficacy requires thorough elucidation, especially across agricultural species beyond traditional model plants. Continued research is essential to optimize AMP formulations and deployment strategies, ensuring safety and effectiveness in agricultural contexts.

### 3.4. Siliforce®, Kalex Zn® and Kalex Cu®

Several commercial formulations have recently been tested against *Xf*, including Siliforce^®^ (a mixture of molybdenum, zinc and silicic acid), Kalex Zn^®^ and Kalex Cu^®^ (with Zn and Cu ions, respectively, together with phosphites).

These three formulations are included in Del Grosso et al.’s study [87] to evaluate their efficacy against *Xf* because they are fertilizers that are already known to improve plant resistance to various stresses and because they may have antimicrobial activity. The strains and subspecies used were *Xf* subsp. *pauca* (strain ST53), *Xf* subsp. *sandyi* (strain CO33) and *Xf* subsp. *multiplex* (strains TOS1 and ESVL) grown on plates with culture medium and disks impregnated with fertilizer solution at different concentrations. After 10 days, the diameter of the inhibition zone was measured, i.e., the part where the bacteria did not grow, because the greater the inhibition zone, the stronger the antibacterial effect. The products tested *in vitro* exhibited different antibacterial activities: the product based on Zn and phosphites was not very sensitive to ST53 but was effective against others, while the product based on Cu and phosphites, not combined with Kalex Zn^®^, inhibited the bacterium at a 6.3% resistance level. Siliforce^®^, on the other hand, was less effective than Kalex Cu^®^, with a resistance of 26.4% at low concentrations and inhibiting bacterial growth only at higher concentrations (4% *v*/*v*). Their bactericidal activity was also distinguished, as Kalex Zn^®^ completely killed the bacterium after 4 h, while Kalex Cu^®^ and the mixture of Kalex Zn^®^ and Kalex Cu^®^ killed it after 2–4 h. In this aspect, Siliforce^®^ was ineffective because it did not kill the bacterium even after 24 h.

*In vivo* trials were carried out with combined treatments of Kalex Zn^®^ and Kalex Cu^®^ with phytopharmaceuticals on *Xf*-infected Cellina di Nardò cv olive trees. The parameters monitored were severity of the disease, area under the curve of disease progression (AUDPC) to analyze the disease trend over time, olive production, oil quality and qPCR analysis for *Xf*. The disease was significantly reduced after treatment, with a disease index increase of only 20% for treated plants compared to 61.5% for untreated plants. Those treated showed a reduction in the bacterial population from May to September 2022 (from 4.88 ± 0.56 Log_10_(CFU/mL) to 4.66 ± 0.63 Log_10_(CFU/mL)) and produced more olives per plant (average of 17 kg per plant in 2021 compared to 2 kg per plant for untreated plants, about 15.7 kg for treated plants in 2022 and about 7.5 kg for untreated plants). The quality of the oil produced by the treated trees was higher, as evidenced by a decrease in acidity (from 0.27% to 0.25%) and peroxides (from 5.4 to 5 meq/kg) [87].

These three formulations have proven promising both *in vitro* and *in vivo* against the *Xf* subspecies studied. The most promising results, however, come from *in vivo* tests carried out on cv Cellina di Nardò olive trees, the reference host for *Xf* subsp. *pauca*, where the combined treatment with Kalex Cu^®^ and Kalex Zn^®^ led to a reduction in bacterial titer and disease severity and also a significant improvement in productivity.

### 3.5. Antibiotics

One experimental strategy for controlling *Xf* is the use of antibiotics, which are being tested for their effectiveness in reducing the bacterial load. Their use, however, raises important questions regarding bacterial resistance and the safety of both humans and the environment, so their use in plants is not permitted in Europe.

However, in order to better understand the susceptibility of *Xf* to different active ingredients, various antibiotics have been tested *in vitro* by Lacava et al. [88] on strains of *Xf* subsp. *pauca* isolated from sweet orange and coffee trees and on a strain of *Xf* subsp. *fastidiosa* isolated from grapevines, demonstrating that the bacterium is sensitive to tetracycline, kanamycin and neomycin but resistant to ampicillin, streptomycin and penicillin-G [88].

In the study by Bleve et al. [89], *Xf* subsp. *pauca* was found to be susceptible to several antibiotics, including ceftriaxone, cephaloridine, cephalotin sodium salt, gentamycin, doxycycline hyclate, oxytetracycline, tetracycline hydrochloride, ampicillin, carbenicillin, penicillin G sodium salt, polymyxin B sulfate and rifampicin. The inhibition zones measured on 5 µg antibiotic disks ranged from 2.0 ± 0.1 mm for gentamycin to 10.0 ± 0.1 mm for ceftriaxone. Conversely, the bacterium was resistant to other antibiotics, such as nystatin, cephalexin hydrate, cycloserine, nalidixic acid, amikacin, kanamycin, kasugamycin, neomycin trisulfate salt hydrate, novobiocin sodium salt, penicillin V potassium salt, vancomycin hydrochloride (from *Streptomyces orientalis*), spiramycin, sulphapyridine, tyrothricin (from *Bacillus aneurinolyticus*), teicoplanin, lincomycin and chloramphenicol.

As demonstrated by these studies, the susceptibility of *Xf* to antibiotics varies among subspecies. However, no data are reported regarding the change in bacterial titer after treatment, making it difficult to accurately assess the antibacterial effect.

## 4. Salt and Metal Compounds

In recent years, some innovative solutions, such as the use of metal-based compounds (e.g., Zn and Cu), have garnered attention due to their biological and chemical properties and their impact on *Xf*. In addition to administering these, other strategies, such as salt-based solutions, have been employed.

### 4.1. Ammonium Chloride

Ammonium salts are water-soluble dissociated ionic compounds. Due to their properties, they find application in various sectors, including agriculture, where they are used as fertilizers [90]. A more common example is ammonium chloride (NH_4_Cl), which represents a source of nitrogen for the soil [91].

To test the efficacy of NH_4_Cl on *in vitro Xf* subsp. *pauca* (strain ST53), it was used at three concentrations. After three days, planktonic cell growth was added to a liquid medium and measured by seeding the sample on agar plates. After six days, biofilm formation and cell adhesion were assessed. After 3 days, bacterial colonies reduced the concentration of NH_4_Cl in the medium, and after 6 days, the amount of planktonic cells also decreased significantly for all tested concentrations. Crystal violet analysis, however, showed a significant decrease in biofilm formation: the optical density (OD) value in the control was 1.6, while in the samples containing NH_4_Cl at 0.25%, 0.5% and 1%, it dropped to 0.8, 0.4 and 0.15, respectively [90].

*In vivo* studies conducted between 2019 and 2020 involved five olive groves with different levels of symptomatology and infection in which four treatments were applied between March and October using NH_4_Cl alone or in combination with biostimulants. No detailed data were reported, but an increase in the production of new vegetation was observed regardless of the NH_4_Cl formulation used, as well as a reduction in symptoms on treated plants. However, no variation in the size of the bacterial population was observed between treated and untreated trees [90].

In conclusion, NH_4_Cl demonstrated a reduction in bacterial titer only *in vitro*. However, in the *in vivo* study conducted on olive trees, despite a reduction in symptoms and vegetative improvement, there was no difference in bacterial titer between treated and untreated plants. This may cast doubt on the long-term efficacy of NH_4_Cl against *Xf*.

### 4.2. Dentamet®

Dentamet^®^ is a biofertilizer containing a mixture of Cu (2% *w/w*) and Zn (4% *w/w*) complexed with citric acid via a fermentation process with antimicrobial properties [92]. Over the years, the effect of Dentamet^®^ against *Xf subsp*. *pauca* has been researched in various ways.

Between June 2016 and September 2017, Scortichini et al. [93] tested the Cu–Zn biofertilizer *in vitro* and *in vivo* on *Xf*-infected Ogliarola salentina and Cellina di Nardò trees, two *Xf*-sensitive cultivars. Analyses with qPCR were performed, and the results showed that in both cultivars, the treated trees had a similar trend in the reduction of *Xf* (approximately 10^2^ CFU equivalents) compared to the untreated trees (10^4^ to 10^5^ CFU equivalents). In terms of symptomatology, the compound reduced the severity of disease symptoms in both varieties, resulting in a good condition at the end of the trial. At the same time, most of the untreated trees were dead [93].

Regarding these two *Xf*-infected olive tree cultivars, the metabolic profiles were analyzed by Girelli et al. [94] using H-NMR spectroscopy, comparing the trees treated for more than a year with the biofertilizer to the untreated trees to assess differences in metabolites within the leaves. In particular, metabolites such as oleuropein in aldehydic form, quinic acid and ligstroside were detected in treated trees, showing an alteration in plant metabolism in response to the treatment [94].

Tatulli et al. [95] also evaluated the efficacy of Dentamet^®^ both *in vitro* and *in vivo*. In the first case, the strains cultured on plates were subsp. *pauca* strain De Donno (isolated from olive trees), subsp. *fastidiosa* strain Temecula1 (isolated from grapevines) and subsp. *multiplex* strain CFBP8416 (isolated from *Polygala myrtifolia*), which were subsequently treated with Dentamet^®^ at different concentrations. In parallel, field trials were also carried out for Ogliarola salentina, Cellina di Nardò and Leccino, where a reduction in symptoms and an average production yield were observed (18–23 kg of olives per tree). In terms of bacterial concentration after the treatments, values ranging from 2.1 to 2.2 × 10^3^ CFU/g were recorded, with no significant differences between cultivars and olive groves, except for Cellina di Nardò in one of the groves, which had a concentration of 4.5 × 10^4^ CFU/g. On the other hand, *in vitro*, it was found that treatment with the biofertilizer had significant effects, as dilutions of 1:10 and 1:50 completely inhibited the growth of the bacterium. Even with a 1:100 dilution, antibacterial activity remained considerable, although less intense. In all dilutions, biofilm production was reduced, particularly in the strain De Donno [95].

In the metabolomic NMR analysis by Girelli et al. [96], the metabolism of Leccino cv was evaluated in comparison to that of Ogliarola salentina and Cellina di Nardò after various treatments. The treatment was administered over six months, and metabolic profiles were evaluated before and after the treatment, showing that Leccino reacted more selectively than the other two cultivars. Furthermore, Leccino demonstrated the ability to maintain and modulate its long-term metabolomic response to the administered treatments. This suggests that *Xf*-resistant cv Leccino responds more effectively to treatments than the other two *Xf*-sensitive cultivars [96].

Treatments can be administered in different ways, and so Hussain et al. [97] examined the differences between spray leaf treatments with Dentamet^®^ and endotherapeutic treatments, highlighting that the best results regarding the separation of metabolic profiles are given by endotherapy injections, but compared to them, the foliar spray treatments are more effective over time. However, to be truly effective, they must be applied in conjunction with appropriate agronomic management practices [97].

Therefore, Dentamet^®^ has proven to be effective both *in vitro* and *in vivo* against *Xf* subspp. *fastidiosa*, *pauca* and *multiplex*, with a significant reduction in biofilm formation, especially for *Xf* subsp. *pauca*, and complete inhibition of bacterial titer *in vitro*. Improvements have also been observed in the field in olive trees, as the treatments have led to an increase in productivity and a reduction in symptoms. However, regarding bacterial titer, *in vivo*, the treatment has had only partial efficacy. Despite the promising results, the effect of Dentamet^®^ also depends greatly on the administration method and agronomic management.

The following table (Table 2) shows in detail the heterogeneity of the experimental design of all molecules tested *in vitro* and *in vivo* against *Xf*.

## 5. Alternative Approaches for Managing *Xf*

In recent years, genetic approaches have made significant contributions to the management of *Xf*. Whole-genome sequencing and CRISPR-based editing tools represent potential foundations for obtaining genetic diagnostic tools that can be easily adapted to the analyzed sample [98]. However, the regulatory and cultural limitations of genetically modified crops remain high, particularly in Europe, as they are heavily regulated [99]. Therefore, an alternative approach may be to study the variability of response among *Xf* host cultivars, with the aim of identifying genotypes that are naturally resistant or tolerant to the infection.

Among olive cultivars, “Cellina di Nardò” and “Leccino” have been extensively studied for their contrasting responses to *Xf* subsp. *pauca*. The former is a widespread variety in Salento (southern Italy) characterized by vigorous trees up to 20 m tall with elongated leaves and early flowering. Olives produced from this cultivar are rich in water, fats and phenolic compounds, including oleuropein [100,101]. The “Leccino” cultivar, on the other hand, is characterized by high vigor, high production even in young plants and good adaptability [102]. The mechanisms of resistance of “Leccino” to *Xf* have not yet been fully defined, but it has been demonstrated that resistance is likely due to the amount of lignin present in the xylem vessels, which slows the movement and spread of the bacterium [103]. 

In the study by De Pascali et al. [104], it was observed that in *Xf*-resistant olive cultivars, such as “Leccino”, higher expression levels were present for genes involved in pathogen stress, such as *LRR-RLK* (leucine-rich repeat genes), *PR* genes related to pathogenesis and genes related to ROS scavenging systems. Genes associated with the response to water stress, such as *PIP2.1* (aquaporin), *DREB* (dehydration responsive element binding) and *DHN* (dehydrin), were more active in the susceptible cultivar “Cellina di Nardò”, but only under drought conditions. When *Xf* was present, these same genes were poorly expressed in *Xf*-susceptible plants, while *DHN* was induced in “Leccino”, despite its lower drought tolerance. This demonstrates that “Leccino” exhibits stable behavior under all stress conditions, while “Cellina” exhibits a marked reduction in water content when infected or subjected to combined stress, thus suggesting that “Leccino” may show resistance to *Xf* due to its poor ability to tolerate water stress, consequently activating alternative defense strategies to defend itself from the pathogen [104].

Studies to evaluate resistance have also been conducted on grapevine cultivars. In fact, Morales-Cruz et al. [105] analyzed specific genetic fragments called R-kmer associated with resistance, demonstrating that *Vitis girdiana* and *Vitis arizonica* (resistant wild species) share R-kmer located in different regions of the genome (chromosomes 14 and 15), suggesting that resistance to Pierce’s disease is likely polygenic (i.e., caused by multiple genomic regions) [105].

Studies such as these reported in the literature confirm that identifying cultivars tolerant and resistant to *Xf* can represent a practical approach for managing the pathogen. In particular, genomic knowledge can aid in the selection of resilient genotypes, offering promising tools for integrated and sustainable management.

## 6. Chemical Control of *Xf* Vector Insects

In addition to chemical strategies aimed directly at *Xf*, it is essential to also intervene on the vector to interrupt the transmission cycle. The control of insect vectors, therefore, is a key element in the integrated management of the disease. Currently, the main vectors of *Xf* are leafhoppers belonging to the genus *Homalodisca*, including *H. vitripennis* and spittlebugs, especially those belonging to the genus *Philaenus*, including *P. spumarius* [106,107]. Spittlebugs are univoltine and overwinter as eggs; current control strategies also allow for targeting the nymphal stages, proving more effective because the nymphs have little ability to move and, therefore, cannot transmit the disease [108], unlike *Homalodisca vitripennis*, which overwinter in the adult form [107].

Chemical control is effective for *Homalodisca*, which has been found to be sensitive to pyrethroids and systemic neonicotinoids [107]. These compounds have a rapid and persistent effect [109], such as the pyrethroid cyfluthrin [110] and the neonicotinoids imidacloprid and acetaprimid [111,112]. Organophosphates such as chlorpyrifos and dimethoate have been shown to be effective in all stages of *H. vitripennis* in contrast to pyrethroids, which have shown high toxicity, especially in the immature stages of the leafhopper [112]. Other control strategies include the use of white kaolin clay sprays, which repel the leafhopper as the particles stick to its wings and legs, preventing egg laying and feeding [109]. Furthermore, other techniques, such as RNA interference (RNAi), defined as sequence-specific silencing of the target gene, are under development and could represent a valid alternative to classic pesticides, potentially making them sustainable [113,114].

Over the years, many insecticides have been evaluated for their efficacy against *P. spumarius*, including, for example, neonicotinoids and pyrethroids. The latter have been shown to provide excellent control against *P. spumarius* nymphs and to have a rapid action, causing high mortality (approximately 90–95%) within 24 h. On the other hand, other products, such as pymetrozine and spirotetramat, do not act effectively against nymphs unless combined with other molecules, such as piperonyl butoxide. This results in a mortality rate of about 30% with pyrethrins alone, increasing to 95% with the combined treatment [115]. Instead, pyrethroids such as deltamethrin and neonicotinoids such as imidacloprid are much more effective against adults of *P. spumarius*, causing mortality rates ranging from 76 to 100%, unlike organophosphorus insecticides, which were found to be less effective, showing mortality rates from 69 to 82% [116]. Molecules such as spinosad, abemectin and sweet orange essential oil, like organophosphorus insecticides, have also shown little persistence; however, unlike organophosphorus insecticides, they have shown immediate effects [117]. The use of pheromones to manipulate insect behavior should be included in insect vector control strategies. Germinara et al. [118] have demonstrated how *P. spumarius* is able to perceive some volatile compounds that modulate intra- and interspecific interactions, including, for example, 2-octanol, 2-decanone, (E)-2-hexenyl acetate and vanillin [118].

Among the control strategies against *P. spumarius*, different methodologies aimed at weed and soil management were also tested to reduce the young of *P. spumarius*. Applications, including shallow ploughing, soil tillage in early winter and in spring, soil tillage only in early winter, sowing of *Hordeum vulgare* L. and *Lolium* spp., the use of herbicides, flame weeding and mulching, have proven effective against the juvenile stages of both spittlebug species [117].

In conclusion, integrated control strategies represent the most effective approach to reducing the population of vectors and limiting the spread of *Xf*; however, new techniques, such as RNAi, may prove to be a more sustainable and effective solution.

## 7. The Fight Against *Xf*: Conclusions and Prospects

All studies reported so far have shown that most molecules exhibit good antibacterial activity against *Xf* and are capable of inhibiting biofilm formation *in vitro*. However, some factors complicate the application of these molecules in real-field conditions. Primarily, some studies are conducted on model plants, such as *N. tabacum*, or characterized by few independent replicates that do not allow for the evaluation of the long-term efficacy of the molecule and its persistence. For this reason, currently, data from research conducted *in vivo* are scarce, considering the importance of the problem. Furthermore, there are few experiments carried out on plants of agricultural interest, such as olive trees, grapevines or almonds. In addition to the control strategies for the bacterium, the management of the insect vectors responsible for *Xf* transmission is of fundamental importance. However, the success of containment strategies can be influenced by the complexity of the vector and its ability to adapt to different environmental conditions.

Furthermore, it is necessary to consider regulatory constraints that prohibit or limit the field use of certain molecules, particularly synthetic ones such as antibiotics and nanoparticles. The repeated and non-integrated application of chemical treatments could favor the development of resistance in both the bacterium and its vectors, necessitating in-depth consideration of the sustainable use of these strategies.

Therefore, the future of *Xf* control should focus on developing integrated containment strategies that combine sound agronomic practices, early diagnostic tools and control strategies for both the bacterium and the vector. Such a holistic approach aims not only to address the immediate challenges posed by *Xf* but also to ensure the sustainability of agricultural systems in the face of evolving plant pathogens. Continued research and collaboration within the agricultural community will be crucial for developing and implementing these innovative strategies, ultimately mitigating the impact of *Xf* on global food security.

## Figures and Tables

**Table 1:** Summary of nanoparticle-based formulations tested against *Xylella fastidiosa*, detailing their mechanisms of action, distinctive physicochemical properties and the supporting literature.

Type of Nanoparticle	Mechanism of Action	Experimental Conditions	Characteristics	Ref.
Silver (Ag) NPs (ARGIRIUM-SUNCs^®)^	Antibacterial activity	*In vitro*	-Small size (1.79 nm) -Diagonal shape -Presence of Ag oxidation states -Negative solvation layer	[49]
Thymol NP	Antimicrobial activity	*In vitro*	-Affects membrane permeability and structure -Are nanoencapsulated to increase bioavailability and improve thymol stability -Gradual release into target cells	[50,51,52,53,54]
Fosetyl–Al NP	Systemic fungicide with antibacterial activity	*In vitro*	Optimization of diffusion at the target site with nanoformulation by sonification to break chemical bonds and reduce particle size	[55,56,57,58]
Zinkicide^®^	Antimicrobial activity	*In vitro* and *in vivo*	-Particle size (4 nm) -Production of reactive oxygen species (ROS) -Accumulation of Zn ions -Lipid peroxidation -Cell membrane disruption	[59,60,61]
Calcium carbonate nanocarriers	Antibacterial activity	*In vitro* and *in vivo*	-Different ways of integrating molecules -They act as nanocarriers of micro and macromolecules	[62,63,64]

**Table 2:** Summary of molecules tested against *Xylella fastidiosa*, highlighting the differences in *in vitro* and *in vivo* effects.

Molecule	Treated Plant		*In Vitro*		*In Vivo*		Ref.
		*Xf* Subsp.	Bacterial Titer Reduction	Biofilm Reduction	Symptom Reduction	Bacterial Titer Reduction	
N-acetylcysteine	Sweet orange	*pauca*	Yes	Yes	Yes	Yes	[16]
	Olive	*pauca*	No	-	Partial	Partial	[23]
		*fastidiosa*	Yes	Yes	-	-	[27,30,31]
Phenolic compounds		*multiplex*	Yes	-	-	-	[27]
	Olive	*pauca*	Yes	Yes	Partial	-	[31]
Oxylipins		*pauca*	-	Yes (dioxygenase) No (lipoxygenase)	-	-	[34,36]
		*fastidiosa* and *multiplex*	-	Yes	-	-	[37]
NuovOlivo^®^	Olive	*pauca*	-	-	Yes	Yes	[38,39]
Menadione and benzethonium chloride	Grapevine	*fastidiosa*	Yes	-	Yes	-	[44]
Ag nanoparticles		*pauca*	Yes	Yes	-	-	[65]
Thymol nanoparticles	Olive	*pauca*	Yes	-	Yes	Yes	[66]
Fosetyl–Al nanocrystals+ chitosan		*fastidiosa*	Yes	Yes	-	-	[68]
		*pauca*	Yes	No	-	-	[68]
Zinkicide^®^	*N. tabacum*	*fastidiosa*	Yes	Yes	Yes	Yes	[71]
	*V. corymbosum*	*multiplex*	Yes	Yes	Yes	Yes	[71]
Calcium carbonate nanocarriers	Olive	*pauca*	Yes	-	No	No	[73]
Antimicrobial peptides	*N. tabacum*	*fastidiosa*, *multiplex* and *pauca*	Yes	Yes	Partial	-	[81,82,83]
	*N. benthamiana*	*fastidiosa*	Yes	Yes	Yes	Yes	[86]
Gomesina	*N. clevelandii*	*pauca*	Yes	No	Yes	No	[85]
Kalex Zn^®^ Kalex Cu^®^	Olive	*pauca*, *sandyi* and *multiplex*	Yes	-	Yes	Yes	[87]
Siliforce^®^		*pauca*, *sandyi* and *multiplex*	Yes	-	-	-	[87]
Ammonium chloride	Olive	*pauca*	Yes	Yes	Yes	No	[90]
Dentamet^®^	Olive	*pauca*, *fastidiosa* and *multiplex*	Yes	Yes	Yes	Partial	[93]

Yes: effect verified; No: effect not verified; **-**: effect not assessed; Partial: effect partially verified.

## Data Availability

No new data were created or analyzed in this study. Data sharing is not applicable to this article.

## Abbreviations

*Xf*	*Xylella fastidiosa*
NAC	N-acetylcysteine
ROS	Reactive oxygen species
CVC	Citrus Variegated Chlorosis
EPS	Exopolysaccharide
MIC	Minimum inhibitory concentration
NP	Nanoparticles
NanoFos	Fosetyl–Al nanocrystals
CH-nanofos	Chitosan–Fosetyl–Al nanocrystals
ZnO	Zinc oxide
ZnK	Zinkicide^®^
PBS	Phosphate-Buffered Saline
AMPs	Antimicrobial peptides
AUDPC	Area Under the Disease Progress Curve
OD	Optical density

## References

[1] Uceda-Campos G., Feitosa-Junior O.R., Santiago C.R.N., Pierry P.M., Zaini P.A., de Santana W.O., Martins-Junior J., Barbosa D., Digiampietri L.A., Setubal J.C. (2022). Comparative Genomics of *Xylella fastidiosa* Explores Candidate Host-Specificity Determinants and Expands the Known Repertoire of Mobile Genetic Elements and Immunity Systems. Microorganisms.

[2] Purcell A.H., Hopkins D.L. (1996). Fastidious Xylem-Limited Bacterial Plant Pathogens. Phytopathol.

[3] Chatterjee S., Almeida R.P.P., Lindow S. (2008). Living in Two Worlds: The Plant and Insect Lifestyles of *Xylella fastidiosa*. Annu. Rev. Phytopathol..

[4] Janse J.D., Obradovic A. (2010). *Xylella fastidiosa*: Its Biology, Diagnosis, Control and Risk. Plant Pathol. J..

[5] Weng L.W., Lin Y.C., Su C.C., Huang C.T., Cho S.T., Chen A.P., Chou S.J., Tsai C.W., Kuo C.H. (2021). Complete Genome Sequence of *Xylella taiwanensis* and Comparative Analysis of Virulence Gene Content With *Xylella fastidiosa*. Front. Microbiol..

[6] Niza B., Coletta-Filho H.D., Merfa M.V., Takita M.A., de Souza A.A. (2015). Differential Colonization Patterns of *Xylella fastidiosa* Infecting Citrus Genotypes. Plant Pathol..

[7] Vanhove M., Retchless A.C., Sicard A., Rieux A., Coletta-filho H.D., La Fuente L.D., Stenger D.C., Almeida R.P.P. (2019). Genomic Diversity and Recombination among *Xylella fastidiosa* Subspecies. Appl Environ Microbiol..

[8] Pérez-Giraldo C., Rodriguez-Benito A., Morán F.J., Hurtado C., Blanco M.T., Gómez-Garcí A.C. (1997). Influence of N-Acetylcysteine on the Formation of Biofilm by *Staphylococcus epidermidis*. J. Antimicrob. Chemother..

[9] Hafez M.M., Aboulwafa M.M., Yassien M.A., Hassouna N.A. (2009). Activity of Some Mucolytics against Bacterial Adherence to Mammalian Cells. Appl. Biochem. Biotechnol..

[10] Giampreti A., Lonati D., Ragghianti B., Ronchi A., Petrolini V.M., Vecchio S., Locatelli C.A. (2016). N-Acetyl-Cysteine as Effective and Safe Chelating Agent in Metal-on-Metal Hip-Implanted Patients: Two Cases. Case Rep. Orthop..

[11] D’Ambrosi R., Ursino N. (2020). N-Acetyl-Cysteine Reduces Blood Chromium and Cobalt Levels in Metal-on-Metal Hip Arthroplasty. Arthroplast. Today.

[12] Aldini G., Altomare A., Baron G., Vistoli G., Carini M., Borsani L., Sergio F. (2018). N-Acetylcysteine as an Antioxidant and Disulphide Breaking Agent: The Reasons Why. Free Radic. Res..

[13] Samuni Y., Goldstein S., Dean O.M., Berk M. (2013). The Chemistry and Biological Activities of N-Acetylcysteine. Biochim. Biophys. Acta-Gen. Subj..

[14] Picchi S.C., De Souza e Silva M., Saldanha L.L., Ferreira H., Takita M.A., Caldana C., de Souza A.A. (2021). GC-TOF/MS-Based Metabolomics Analysis to Investigate the Changes Driven by N-Acetylcysteine in the Plant-Pathogen *Xanthomonas citri* subsp. *citri*. Sci. Rep..

[15] Dinicola S., De Grazia S., Carlomagno G., Pintucci J.P. (2014). N-Acetylcysteine as Powerful Molecule to Destroy Bacterial Biofilms. A Systematic Review. Eur. Rev. Med. Pharmacol. Sci..

[16] Muranaka L.S., Giorgiano T.E., Takita M.A., Forim M.R., Silva L.F.C., Coletta-Filho H.D., Machado M.A., de Souza A.A. (2013). N-Acetylcysteine in Agriculture, a Novel Use for an Old Molecule: Focus on Controlling the Plant-Pathogen *Xylella fastidiosa*. PLoS ONE.

[17] Da Silva A.M., Murillo D.M., Anbumani S., von Zuben A.A., Cavalli A., Obata H.T., Fischer E.R., de Souza e Silva M., Bakkers E., Souza A.A. (2024). N-Acetylcysteine Effects on Extracellular Polymeric Substances of *Xylella fastidiosa*: A Spatiotemporal Investigation with Implications for Biofilm Disruption. Int. J. Antimicrob. Agents.

[18] Helenius J., Heisenberg C.P., Gaub H.E., Muller D.J. (2008). Single-Cell Force Spectroscopy. J. Cell Sci..

[19] Cattò C., De Vincenti L., Cappitelli F., D’attoma G., Saponari M., Villa F., Forlani F. (2019). Non-Lethal Effects of N-Acetylcysteine on *Xylella fastidiosa* Strain de Donno Biofilm Formation and Detachment. Microorganisms.

[20] Wise J.C., VanWoerkom A.H., Aćimović S.G., Sundin G.W., Cregg B.M., Vandervoort C. (2014). Trunk Injection: A Discriminating Delivering System for Horticulture Crop IPM. Entomol. Ornithol. Herpetol. Curr. Res..

[21] Archer L., Crane J.H., Albrecht U. (2024). Correction: Archer et al. Trunk Injection as a Tool to Deliver Plant Protection Materials—An Overview of Basic Principles and Practical Considerations. *Horticulturae*
**2022**, *8*, 552. Horticulturae.

[22] Picchi S.C., Rebelatto D., Martins P.M.M., Blumer S., Mesquita G.L., Hippler F.W.R., Mattos D., Boaretto R.M., Machado M.A., Takita M.A. (2024). N-Acetylcysteine Absorption and Its Potential Dual Effect Improve Fitness and Fruit Yield in *Xylella fastidiosa* Infected Plants. Pest Manag. Sci..

[23] Boscia D., Saponari M. (2019). Recent advances on the control of *Xylella fastidiosa* and its vectors in olive groves: State of the art from the ongoing Europe’s Horizon 2020 Research Program. J. Plant Pathol..

[24] Boudet A.M. (2007). Evolution and Current Status of Research in Phenolic Compounds. Phytochemistry.

[25] Obied H.K., Allen M.S., Bedgood D.R., Prenzler P.D., Robards K., Stockmann R. (2005). Bioactivity and Analysis of Biophenols Recovered from Olive Mill Waste. J. Agric. Food Chem..

[26] Azaizeh H., Tafesh A., Najami N., Jadoun J., Halahlih F., Riepl H. (2011). Synergistic Antibacterial Effects of Polyphenolic Compounds from Olive Mill Wastewater. Evid.-Based Complement. Altern. Med..

[27] Maddox C.E., Laur L.M., Tian L. (2010). Antibacterial Activity of Phenolic Compounds against the Phytopathogen *Xylella fastidiosa*. Curr. Microbiol..

[28] Taguri T., Tanaka T., Kouno I. (2006). Antibacterial Spectrum of Plant Polyphenols and Extracts Depending upon Hydroxyphenyl Structure. Biol. Pharm. Bull..

[29] Evans S.M., Cowan M.M. (2016). Plant Products as Antimicrobial Agents. Cosmet. Drug Microbiol..

[30] Lee S.A., Wallis C.M., Rogers E.E., Burbank L.P. (2020). Grapevine Phenolic Compounds Influence Cell Surface Adhesion of *Xylella fastidiosa* and Bind to Lipopolysaccharide. PLoS ONE.

[31] Vizzarri V., Ienco A., Benincasa C., Perri E., Pucci N., Cesari E., Novellis C., Rizzo P., Pellegrino M., Zaffina F. (2023). Phenolic Extract from Olive Leaves as a Promising Endotherapeutic Treatment against *Xylella fastidiosa* in Naturally Infected Olea Europaea (Var. Europaea) Trees. Biology.

[32] De Micco V., Balzano A., Wheeler E.A., Baas P. (2016). Tyloses and Gums: A Review of Structure, Function and Occurrence of Vessel Occlusions. IAWA J..

[33] Mumtaz F., Zubair M., Khan F., Niaz K., Sanches Silva A., Nabavi S.F., Saeedi M., Nabavi S.M. (2020). Chapter 22. Analysis of Plants Lipids. Recent Advances in Natural Products Analysis.

[34] Scala V., Pucci N., Salustri M., Modesti V., L’Aurora A., Scortichini M., Zaccaria M., Momeni B., Reverberi M., Loreti S. (2020). *Xylella fastidiosa* subsp. *pauca* and Olive Produced Lipids Moderate the Switch Adhesive versus Non-Adhesive State and Viceversa. PLoS ONE.

[35] Scala V., Reverberi M., Salustri M., Pucci N., Modesti V., Lucchesi S., Loreti S. (2018). Lipid Profile of *Xylella fastidiosa* subsp. *pauca* Associated with the Olive Quick Decline Syndrome. Front. Microbiol..

[36] Scala V., Pucci N., Salustri M., Modesti V., L’Aurora V., Scortichini M., Zaccaria M., Momeni B., Reverberi M., Loreti S. (2019). Bacterial and Plant Produced Lipids Can Exacerbate the Olive Quick Decline Syndrome Caused by *Xylella*. Sustainability.

[37] Scala V., Salustri M., Merfa M.V., Beccaccioli M., Lascala L., De La Fuente L., Reverberi M. (2025). *Xad*A-like Adhesin XADA2 Regulates Biofilm Formation in *X. fastidiosa* subsp. *fastidiosa* Putatively by Engaging Oleic-Acid Derived Oxylipins. Mol. Biol. Rep..

[38] Bruno G.L., Cariddi C., Botrugno L. (2021). Exploring a Sustainable Solution to Control *Xylella fastidiosa* subsp. *pauca* on Olive in the Salento Peninsula, Southern Italy. Crop Prot..

[39] Bruno G.L. (2024). Coexistence between *Xylella fastidiosa* subsp. *pauca* and Susceptible Olive Plants in the Salento Peninsula (Southern Italy). Agronomy.

[40] Corey E.J. (1968). Stereospecific Total Synthesis of the *dl*-C_18_ Cecropia Juvenile Hormone. J. Am. Chem. Soc..

[41] Robin D.C., Marchand P.A. (2019). Evolution of the Biocontrol Active Substances in the Framework of the European Pesticide Regulation (EC) No. 1107/2009. Pest Manag. Sci..

[42] European Food Safety Authority (2014). Scientific Opinion on the Safety and Efficacy of Vitamin K3 (Menadione Sodium Bisulphite and Menadione Nicotinamide Bisulphite) as a Feed Additive for All Animal Species. EFSA J..

[43] Zhao D., Zhang Y., Jin Z., Bai R., Wang J., Wu L., He Y. (2024). Benzalkonium Chloride and Benzethonium Chloride Effectively Reduce Spore Germination of Ginger Soft Rot Pathogens: *Fusarium solani* and *Fusarium oxysporum*. J. Fungi.

[44] Zhang S., Jain M., Fleites L.A., Rayside P.A., Gabriel D.W. (2019). Identification and Characterization of Menadione and Benzethonium Chloride as Potential Treatments of Pierce’s Disease of Grapevines. Phytopathology.

[45] Singh Sekhon B. (2014). Nanotechnology in Agri-Food Production: An Overview. Nanotechnol. Sci. Appl..

[46] Kookana R.S., Boxall A.B.A., Reeves P.T., Ashauer R., Beulke S., Chaudhry Q., Cornelis G., Fernandes T.F., Gan J., Kah M. (2014). Nanopesticides: Guiding Principles for Regulatory Evaluation of Environmental Risks. J. Agric. Food Chem..

[47] Sundin G.W., Castiblanco L.F., Yuan X., Zeng Q., Yang C.H. (2016). Bacterial Disease Management: Challenges, Experience, Innovation and Future Prospects: Challenges in Bacterial Molecular Plant Pathology. Mol. Plant Pathol..

[48] Li Y., Zhang P., Li M., Shakoor N., Adeel M., Zhou P., Guo M., Jiang Y., Zhao W., Lou B.Z. (2023). Application and Mechanisms of Metal-Based Nanoparticles in the Control of Bacterial and Fungal Crop Diseases. Pest Manag. Sci..

[49] Angelini G., Scotti L., Aceto A., Gasbarri C. (2019). Silver Nanoparticles as Interactive Media for the Azobenzenes Isomerization in Aqueous Solution: From Linear to Stretched Kinetics. J. Mol. Liq..

[50] Chavan P.S., Tupe S.G. (2014). Antifungal Activity and Mechanism of Action of Carvacrol and Thymol against Vineyard and Wine Spoilage Yeasts. Food Control.

[51] Trombetta D., Castelli F., Sarpietro M.G., Venuti V., Cristani M., Daniele C., Saija A., Mazzanti G., Bisignano G. (2005). Mechanisms of Antibacterial Action of Three Monoterpenes. Antimicrob. Agents Chemother..

[52] Zikeli F., Vinciguerra V., Sennato S., Scarascia Mugnozza G., Romagnoli M. (2020). Preparation of Lignin Nanoparticles with Entrapped Essential Oil as a Bio-Based Biocide Delivery System. ACS Omega.

[53] Mattos B.D., Tardy B.L., Pezhman M., Kämäräinen T., Linder M., Schreiner W.H., Magalhães W.L.E., Rojas O.J. (2018). Controlled Biocide Release from Hierarchically-Structured Biogenic Silica: Surface Chemistry to Tune Release Rate and Responsiveness. Sci. Rep..

[54] Medina E., Caro N., Abugoch L., Gamboa A., Díaz-Dosque M., Tapia C. (2019). Chitosan Thymol Nanoparticles Improve the Antimicrobial Effect and the Water Vapour Barrier of Chitosan-Quinoa Protein Films. J. Food Eng..

[55] Suresh Kumar R.S., Shiny P.J., Anjali C.H., Jerobin J., Goshen K.M., Magdassi S., Mukherjee A., Chandrasekaran N. (2013). Distinctive Effects of Nano-Sized Permethrin in the Environment. Environ. Sci. Pollut. Res..

[56] Yearla S.R., Padmasree K. (2016). Exploitation of Subabul Stem Lignin as a Matrix in Controlled Release Agrochemical Nanoformulations: A Case Study with Herbicide Diuron. Environ. Sci. Pollut. Res..

[57] Koshani R., Jafari S.M. (2019). Ultrasound-Assisted Preparation of Different Nanocarriers Loaded with Food Bioactive Ingredients. Adv. Colloid Interface Sci..

[58] Mallakpour S., Abdolmaleki A., Tabesh F. (2018). Ultrasonic-Assisted Manufacturing of New Hydrogel Nanocomposite Biosorbent Containing Calcium Carbonate Nanoparticles and Tragacanth Gum for Removal of Heavy Metal. Ultrason. Sonochem..

[59] Naranjo E., Merfa V.M., Santra S., Ozcan A., Johnson E., Cobine A.P., De La Fuente L. (2020). Crossm Activity against Liberibacter crescens in Batch Cultures and In Batch Cultures and in Microfluidic Chambers Simulating Plant Vascular Systems. Appl. Environ. Microbiol..

[60] Graham J.H., Johnson E.G., Myers M.E., Young M., Rajasekaran P., Das S., Santra S. (2016). Potential of Nano-Formulated Zinc Oxide for Control of Citrus Canker on Grapefruit Trees. Plant Dis..

[61] Sirelkhatim A., Mahmud S., Seeni A., Kaus N.H.M., Ann L.C., Bakhori S.K.M., Hasan H., Mohamad D. (2015). Review on Zinc Oxide Nanoparticles: Antibacterial Activity and Toxicity Mechanism. Nano-Micro Lett..

[62] Peters R.J.B., Bouwmeester H., Gottardo S., Amenta V., Arena M., Brandhoff P., Marvin H.J.P., Mech A., Moniz F.B., Pesudo L.Q. (2016). Nanomaterials for Products and Application in Agriculture, Feed and Food. Trends Food Sci. Technol..

[63] Chen H., Yada R. (2011). Nanotechnologies in Agriculture: New Tools for Sustainable Development. Trends Food Sci. Technol..

[64] Tapia-Hernández J.A., Torres-Chávez P.I., Ramírez-Wong B., Rascón-Chu A., Plascencia-Jatomea M., Barreras-Urbina C.G., Rangel-Vázquez N.A., Rodríguez-Félix F. (2015). Micro- and Nanoparticles by Electrospray: Advances and Applications in Foods. J. Agric. Food Chem..

[65] Orfei B., Moretti C., Loreti S., Tatulli G., Onofri A., Scotti L., Aceto A., Buonaurio R. (2023). Silver Nanoclusters with Ag^2+/3+^ Oxidative States Are a New Highly Effective Tool against Phytopathogenic Bacteria. Appl. Microbiol. Biotechnol..

[66] Baldassarre F., Schiavi D., Ciarroni S., Tagliavento V., De Stradis A., Vergaro V., Suranna G.P., Balestra G.M., Ciccarella G. (2023). Thymol-Nanoparticles as Effective Biocides against the Quarantine Pathogen *Xylella fastidiosa*. Nanomaterials.

[67] Cagnarini C., De Angelis P., Liberati D., Valentini R., Falanga V., Valentini F., Dongiovanni C., Carrieri M., Chiriacò M.V. (2025). Physiological Response of Olive Trees Under *Xylella fastidiosa* Infection and Thymol Therapy Monitored Through Advanced IoT Sensors. Plants.

[68] Baldassarre F., Tatulli G., Vergaro V., Mariano S., Scala V., Nobile C., Pucci N., Dini L., Loreti S., Ciccarella G. (2020). Sonication-Assisted Production of Fosetyl-Al Nanocrystals: Investigation of Human Toxicity and in vitro Antibacterial Efficacy against *Xylella fastidiosa*. Nanomaterials.

[69] Tatulli G., Baldassarre F., Schiavi D., Tacconi S., Cognigni F., Costantini F., Balestra G.M., Dini L., Pucci N., Rossi M. (2024). Chitosan-Coated Fosetyl-Al Nanocrystals’ Efficacy on *Nicotiana Tabacum* Colonized by *Xylella Fastidiosa*. Phytopathology.

[70] Hernández-Montelongo J., Nascimento V.F., Murillo D., Taketa T.B., Sahoo P., De Souza A.A., Beppu M.M., Cotta M.A. (2016). Nanofilms of Hyaluronan/Chitosan Assembled Layer-by-Layer: An Antibacterial Surface for *Xylella Fastidiosa*. Carbohydr. Polym..

[71] Shantharaj D., Naranjo E., Merfa M.V., Cobine P.A., Santra S., De La Fuente L. (2023). Zinc Oxide-Based Nanoformulation Zinkicide Mitigates the Xylem-Limited Pathogen *Xylella fastidiosa* in Tobacco and Southern Highbush Blueberry. Plant Dis..

[72] Boyjoo Y., Pareek V.K., Liu J. (2014). Synthesis of Micro and Nano-Sized Calcium Carbonate Particles and Their Applications. J. Mater. Chem. A.

[73] Baldassarre F., De Stradis A., Altamura G., Vergaro V., Citti C., Cannazza G., Capodilupo A.L., Dini L., Ciccarella G. (2020). Application of Calcium Carbonate Nanocarriers for Controlled Release of Phytodrugs against *Xylella fastidiosa* Pathogen. Pure Appl. Chem..

[74] Schwechheimer C., Sullivan C.J., Kuehn M.J. (2013). Envelope Control of Outer Membrane Vesicle Production in Gram-Negative Bacteria. Biochemistry.

[75] Seo M.D., Won H.S., Kim J.H., Mishig-Ochir T., Lee B.J. (2012). Antimicrobial Peptides for Therapeutic Applications: A Review. Molecules.

[76] Huan Y., Kong Q., Mou H., Yi H. (2020). Antimicrobial Peptides: Classification, Design, Application and Research Progress in Multiple Fields. Front. Microbiol..

[77] Ahmed T.A.E., Hammami R. (2019). Recent Insights into Structure–Function Relationships of Antimicrobial Peptides. J. Food Biochem..

[78] Zhu S., Sani M.A., Separovic F. (2018). Interaction of Cationic Antimicrobial Peptides from Australian Frogs with Lipid Membranes. Pept. Sci..

[79] Alves D., Olívia Pereira M. (2014). Mini-Review: Antimicrobial Peptides and Enzymes as Promising Candidates to Functionalize Biomaterial Surfaces. Biofouling.

[80] Altman H., Steinberg D., Porat Y., Mor A., Fridman D., Friedman M., Bachrach G. (2006). In Vitro Assessment of Antimicrobial Peptides as Potential Agents against Several Oral Bacteria. J. Antimicrob. Chemother..

[81] El Handi K., Sabri M., Valentini F., De Stradis A., Achbani E.H., Hafidi M., El Moujabber M., Elbeaino T. (2022). Exploring Active Peptides with Antimicrobial Activity In Planta against *Xylella fastidiosa*. Biology.

[82] Baró A., Badosa E., Montesinos L., Feliu L., Planas M., Montesinos E., Bonaterra A. (2020). Screening and Identification of BP100 Peptide Conjugates Active against *Xylella fastidiosa* Using a Viability-QPCR Method. BMC Microbiol..

[83] Moll L., Badosa E., Planas M., Feliu L., Montesinos E., Bonaterra A. (2021). Antimicrobial Peptides With Antibiofilm Activity Against *Xylella fastidiosa*. Front. Microbiol..

[84] Li Z.T., Gray D.J. (2003). Effect of Five Antimicrobial Peptides on the Growth of *Agrobacterium Tumefaciens*, *Escherichia Coli* and *Xylella Fastidiosa*. Vitis.

[85] Fogaça A.C., Zaini P.A., Wulff N.A., Da Silva P.I.P., Fázio M.A., Miranda A., Daffre S., Da Silva A.M. (2010). Effects of the Antimicrobial Peptide Gomesin on the Global Gene Expression Profile, Virulence and Biofilm Formation of *Xylella fastidiosa*. FEMS Microbiol. Lett..

[86] Baró A., Saldarelli P., Saponari M., Montesinos E., Montesinos L. (2022). Nicotiana Benthamiana as a Model Plant Host for *Xylella fastidiosa*: Control of Infections by Transient Expression and Endotherapy with a Bifunctional Peptide. Front. Plant Sci..

[87] Del Grosso C., Saponari M., Saldarelli P., Palmieri D., Altamura G., Kubaa R.A., De Curtis F., Lima G. (2025). Use of commercial fertilizers in an IPDM protocol to mitigate Olive Quick Decline Syndrome caused by *Xylella fastidiosa* subsp. *pauca* in Southern Italy. Plant Dis..

[88] Lacava P.T., Araújo W.L., Maccheroni W., Azevedo J.L. (2001). RAPD Profile and Antibiotic Susceptibility of *Xylella fastidiosa*, Causal Agent of Citrus Variegated Chlorosis. Lett. Appl. Microbiol..

[89] Bleve G., Gallo A., Altomare C., Vurro M., Maiorano G., Cardinali A., D’Antuono I., Marchi G., Mita G. (2018). In vitro Activity of Antimicrobial Compounds against *Xylella fastidiosa*, the Causal Agent of the Olive Quick Decline Syndrome in Apulia (Italy). FEMS Microbiol. Lett..

[90] Dongiovanni C., Fumarola G., Zicca S., Surano A., Di Carolo M., Datome G. In vitro and in vivo Effects of Ammonium Chloride on *Xylella fastidiosa* subsp. *pauca* Infecting Olives. Proceedings of the 3rd European Conference on *Xylella fastidiosa* and XF-ACTORS final meeting.

[91] Stefan-Kharicha M., Kharicha A., Mogeritsch J., Wu M., Ludwig A. (2018). Review of Ammonium Chloride-Water Solution Properties. J. Chem. Eng. Data.

[92] Scortichini M., Loreti S., Pucci N., Scala V., Tatulli G., Verweire D., Oehl M., Widmer U., Codina J.M., Hertl P. (2021). Progress towards Sustainable Control of *Xylella fastidiosa* subsp. *pauca* in Olive Groves of Salento (Apulia, Italy). Pathogens.

[93] Scortichini M., Chen J., De Caroli M., Dalessandro G., Pucci N., Modesti V., L’Aurora A., Petriccione M., Zampella L., Mastrobuoni F. (2018). A Zinc, Copper and Citric Acid Biocomplex Shows Promise for Control of *Xylella fastidiosa* subsp. *pauca* in Olive Trees in Apulia Region (Southern Italy). Phytopathol. Mediterr..

[94] Girelli C.R., Angilè F., Del Coco L., Migoni D., Zampella L., Marcelletti S., Cristella N., Marangi P., Scortichini M., Fanizzi F.P. (2019). 1H-NMR Metabolite Fingerprinting Analysis Reveals a Disease Biomarker and a Field Treatment Response in *Xylella fastidiosa* subsp. *pauca*-Infected Olive Trees. Plants.

[95] Tatulli G., Modesti V., Pucci N., Scala V., L’aurora A., Lucchesi S., Salustri M., Scortichini M., Loreti S. (2021). Further in vitro Assessment and Mid-Term Evaluation of Control Strategy of *Xylella fastidiosa* subsp. *pauca* in Olive Groves of Salento (Apulia, Italy). Pathogens.

[96] Girelli C.R., Del Coco L., Angilè F., Scortichini M., Fanizzi F.P. (2021). Olive Cultivars Susceptible or Tolerant to *Xylella fastidiosa* subsp. *pauca* Exhibit Mid-Term Different Metabolomes upon Natural Infection or a Curative Treatment. Plants.

[97] Hussain M., Girelli C.R., Verweire D., Oehl M.C., Avendaño M.S., Scortichini M., Fanizzi F.P. (2023). 1H-NMR Metabolomics Study after Foliar and Endo-Therapy Treatments Of *Xylella fastidiosa* subsp. *pauca* Infected Olive Trees: Medium Time Monitoring of Field Experiments. Plants.

[98] Raffini F., Bertorelle G., Biello R., D’Urso G., Russo D., Bosso L. (2020). From Nucleotides to Satellite Imagery: Approaches to Identify and Manage the Invasive Pathogen *Xylella* Fastidiosa and Its Insect Vectors in Europe. Sustainability.

[99] Chartois M., Mesmin X., Quiquerez I., Borgomano S., Farigoule P., Pierre É., Thuillier J.M., Streito J.C., Casabianca F., Hugot L. (2023). Environmental Factors Driving the Abundance of *Philaenus Spumarius* in Mesomediterranean Habitats of Corsica (France). Sci. Rep..

[100] Aprile A., Negro C., Sabella E., Luvisi A., Nicolì F., Nutricati E., Vergine M., Miceli A., Blando F., De Bellis L. (2019). Antioxidant Activity and Anthocyanin Contents in Olives (CV Cellina Di Nardò) during Ripening and after Fermentation. Antioxidants.

[101] Ryan D., Robards K. (1998). Phenolic Compounds in Olives. Analyst.

[102] Proietti P., Nasini L., Reale L., Caruso T., Ferranti F. (2015). Productive and Vegetative Behavior of Olive Cultivars in Super High-Density Olive Grove. Sci. Agric..

[103] Sabella E., Luvisi A., Aprile A., Negro C., Vergine M., Nicolì F., Miceli A., De Bellis L. (2018). *Xylella Fastidiosa* Induces Differential Expression of Lignification Related-Genes and Lignin Accumulation in Tolerant Olive Trees Cv. Leccino. J. Plant Physiol..

[104] De Pascali M., Vergine M., Sabella E., Aprile A., Nutricati E., Nicolì F., Buja I., Negro C., Miceli A., Rampino P. (2019). Molecular Effects of *Xylella Fastidiosa* and Drought Combined Stress in Olive Trees. Plants.

[105] Morales-Cruz A., Aguirre-Liguori J., Massonnet M., Minio A., Zaccheo M., Cochetel N., Walker A., Riaz S., Zhou Y., Cantu D. (2023). Multigenic Resistance to *Xylella Fastidiosa* in Wild Grapes (Vitis Sps.) and Its Implications within a Changing Climate. Commun. Biol..

[106] Hopkins D.L., Purcell A.H. (2002). Xylella fastidiosa: Cause of Pierce’s Disease of Grapevine and Other Emergent Diseases. Plant Dis..

[107] Mårtensson A. (2007). Need for Protective Measures to Combat Potential Outbreaks of *Homalodisca coagulata* and Pierce’s Disease in European Viticulture. Acta Agric. Scand. Sect. B Soil Plant Sci..

[108] Cornara D., Bosco D., Fereres A. (2018). Philaenus Spumarius: When an Old Acquaintance Becomes a New Threat to European Agriculture. J. Pest Sci..

[109] Purcell A., Feil H. (2001). Glassy-Winged Sharpshooter. Pestic. Outlook.

[110] Bethke J.A., Blua M.J., Redak R.A. (2001). Effect of Selected Insecticides on *Homalodisca coagulata* (Homoptera: Cicadellidae) and Transmission of Oleander Leaf Scorch in a Greenhouse Study. J. Econ. Entomol..

[111] Rathé A.A., Pilkington L.J., Gurr G.M., Hoddle M.S., Daugherty M.P., Constable F.E., Luck J.E., Powell K.S., Fletcher M.J., Edwards O.R. (2012). Incursion Preparedness: Anticipating the Arrival of an Economically Important Plant Pathogen *Xylella fastidiosa* Wells (Proteobacteria: Xanthomonadaceae) and the Insect Vector *Homalodisca vitripennis* (Germar) (Hemiptera: Cicadellidae) in Australia. Aust. J. Entomol..

[112] Prabhaker N., Castle S.J., Toscano N.C. (2006). Susceptibility of Immature Stages of *Homalodisca coagulata* (Hemiptera: Cicadellidae) to Selected Insecticides. J. Econ. Entomol..

[113] Mamta B., Rajam M.V. (2017). RNAi Technology: A New Platform for Crop Pest Control. Physiol. Mol. Biol. Plants.

[114] Willow J., Smagghe G. (2025). RNAi Applications toward Environmentally Sustainable Food Security. Curr. Opin. Environ. Sci. Health.

[115] Dáder B., Viñuela E., Moreno A., Plaza M., Garzo E., Del Estal P., Fereres A. (2019). Sulfoxaflor and Natural Pyrethrin with Piperonyl Butoxide Are Effective Alternatives to Neonicotinoids against Juveniles of *Philaenus spumarius*, the European Vector of *Xylella fastidiosa*. Insects.

[116] Dongiovanni C., Altamura G., Di Carolo M., Fumarola G., Saponari M., Cavalieri V. (2018). Evaluation of Efficacy of Different Insecticides Against *Philaenus spumarius* L., Vector of *Xylella fastidiosa* in Olive Orchards in Southern Italy, 2015–17. Arthropod Manag. Tests.

[117] Dongiovanni C., Di Carolo M., Fumarola G., Tauro D., Altamura G., Cavalieri V. (2018). Evaluation of Insecticides for the Control of Juveniles of *Philaenus Spumarius* L., 2015–2017. Arthropod Manag. Tests.

[118] Germinara G.S., Ganassi S., Pistillo M.O., Di Domenico C., De Cristofaro A., Di Palma A.M. (2017). Antennal Olfactory Responses of Adult Meadow Spittlebug, *Philaenus spumarius*, to Volatile Organic Compounds (VOCs). PLoS ONE.

